# Artificial Environments for the Co-Translational Stabilization of Cell-Free Expressed Proteins

**DOI:** 10.1371/journal.pone.0056637

**Published:** 2013-02-22

**Authors:** Lei Kai, Volker Dötsch, Ralf Kaldenhoff, Frank Bernhard

**Affiliations:** 1 Centre for Biomolecular Magnetic Resonance, Institute for Biophysical Chemistry, Goethe-University of Frankfurt/Main, Frankfurt/Main, Germany; 2 Institute of Botany, Applied Plant Science, Darmstadt University of Technology, Darmstadt, Germany; Centro Nacional de Biotecnologia - CSIC, Spain

## Abstract

An approach for designing individual expression environments that reduce or prevent protein aggregation and precipitation is described. Inefficient folding of difficult proteins in unfavorable translation environments can cause significant losses of overexpressed proteins as precipitates or inclusion bodies. A number of chemical chaperones including alcohols, polyols, polyions or polymers are known to have positive effects on protein stability. However, conventional expression approaches can use such stabilizing agents only post-translationally during protein extraction and purification. Proteins that already precipitate inside of the producer cells cannot be addressed. The open nature of cell-free protein expression systems offers the option to include single chemicals or cocktails of stabilizing compounds already into the expression environment. We report an approach for systematic screening of stabilizers in order to improve the solubility and quality of overexpressed proteins co-translationally. A comprehensive list of representative protein stabilizers from the major groups of naturally occurring chemical chaperones has been analyzed and their concentration ranges tolerated by cell-free expression systems have been determined. As a proof of concept, we have applied the method to improve the yield of proteins showing instability and partial precipitation during cell-free synthesis. Stabilizers that co-translationally improve the solubility and functional folding of human glucosamine 6-phosphate N-acetyltransferase have been identified and cumulative effects of stabilizers have been studied.

## Introduction

Newly synthesized proteins are at great risk of aberrant folding already inside the cellular environment. Formation of aggregates or inclusion bodies composed out of denatured proteins is commonly observed in particular during overexpression of proteins [1]. In addition, protein denaturation could result from degradation mechanisms such as deamidation or oxidation. While refolding can sometimes help to rescue proteins, often high amounts of sample are lost and not useful for further applications. Living cells can support the stability of proteins by a number of organic substances known also as chemical chaperones [2]. Upon recombinant protein production, such chemicals are unfortunately only of limited value as access to the inner cell compartment in conventional cell-based expression systems is restricted. Increasing intracellular concentrations of stabilizers by e.g. inducing specific solute transporters requires strong impacts such as osmotic shocks which could cause dramatic changes in cell physiology and expression patterns [3], [4]. Stabilization strategies are therefore usually confined to manipulations of growth conditions or to attempts of post-translational stabilization during protein extraction, when significant protein precipitation might already have occurred. Cell-free (CF) expression systems offer the new option to support the stability of expressed proteins already co-translationally with a wide and diverse range of additives, while on the other hand being relatively sensitive to manipulations of reaction conditions such as incubation temperature. The open nature of CF reactions allows to supply any tolerated chemical directly into the protein expression environment [5]. Production protocols for unstable and difficult proteins can therefore be individually designed and stabilizers or mixtures thereof can be adjusted according to specific requirements.

Protein stabilizing agents comprise a wide range of chemicals including alcohols and molecular crowding agents such as polyethylenglycols (PEG). Many organisms accumulate small organic molecules in stress situations, which are generally called osmolytes [6], [7]. Those solutes act as chemical chaperones in the cell by preventing protein unfolding and improving protein thermostability. Major groups of osmolytes are polyols, amino acids, polyions or urea [2]. Prominent examples are the synthesis of betaine or trehalose in *E. coli*, glycerol in *Saccharomyces cerevisiae* and generally a number of different polyols and amino acid derivatives in yeasts and plants [7]. Hyperthermophilic microorganisms accumulate organic solutes such as betaine, ectoine or trehalose in high concentrations while responding to heat stress [8], [9]. The intracellular concentration of some of these compounds can even reach molar levels dependent on medium osmolality and growth conditions [10].

CF reactions are ideal for screening experiments and have been applied for the expression of target libraries [11]–[13], protein evolution [14] or drug screening [15]. We have established a process based on extracts of *E. coli* cells and on the batch configuration that allows the screening of chemical chaperones. The tolerated concentration ranges of all additives were determined in linear screening schemes and by using shifted green fluorescent protein (sGFP) as expression monitor. Additives showing positive effects on sGFP fluorescence were then further analyzed in linear or in correlated screening schemes for their effects on two unstable proteins. The screening process for co-translational protein stabilization was exemplified with the human glucosamine 6-phosphate N-acetyltransferase (GNA1) and with the halogenase domain of the fungal CurA polyketide synthetase [16]. Improved solubility of the two proteins was in particular monitored with choline and L-arginine and cumulative effects of selected compounds were analyzed in correlated screens. The established process could provide guidelines and options for the preparative scale production of unstable proteins as well as for exploiting the stabilizing role of osmolytes for biotechnology purposes.

## Materials and Methods

### Chemicals

PEG 6000 was obtained from Applichem (Darmstadt, Germany). All other chemicals were from Sigma-Aldrich (Taufkirchen, Germany) and obtained at highest purity.

### DNA Templates

Shifted green fluorescence protein (sGFP) was cloned into the pIVEX 2.3d vector and expressed with a C-terminal poly(His)_10_ tag using restriction free cloning. The coding region of human GNA1 (GenBank access code BC012179.1) was first cloned into the vector pET21a. A C-terminal fusion of sGFP to GNA1 was then constructed by restriction free cloning. The forward primer had a 24 base overlap complementary to the 5′ end of the desired insertion site of the vector and followed by a start codon and 20–25 bases of the 5′ end of GNA1 coding sequence. The reverse primer annealed to the vector with 24 bases complementary to the 3′ end of the insertion site. A pair of primers was furthermore designed in order to fuse the TEV-sGFP gene sequence after the GNA1 gene sequence (Table 1). The CurA halogenase domain was cloned into the vector pET28b (Merck Bioscience, Darmstadt, Germany) and expressed with an N-terminal His_6_-tag. The native protein sequence covers the amino acids 1599 to 1930 of CurA according to the sequence accessible at NCBI (GenBank accession code: AAT70096.1). DNA templates used for CF expression were transformed into *E. coli* strain DH5α and isolated by standard plasmid purification kits (Macherey-Nagel, Düren, Germany).

**Table 1:** Construction of DNA templates.

Construct	Vector	Modification	Primer sequence^1^
sGFP	pIVEX2.3d	C-poly(His)_10_	F: TTTTGTTTAACTTTAAGAAGGAGATATAC ATATGAGCAAAGGAGAAGAACTTTTCAC
			R: GTGGTGGTGGTGGTGGTGGTGGTGGGATC CCTCGAGTGCGGCCGCAAGCTTTTTGTA
GNA1	pET21a	C-poly(His)_6_	F: CGCGGATCCATGAAACCTGATGAAACTCCT
			R: CCGCTCGAGCTTTAGAAACCTCCGACACA
GNA1-sGFP	pET21a	C-poly(His)_10_ TEV cleavage	F: CTACATGTGTCGGAGGTTTCTAAAGGGCG AAAACCTGTACTTCCAGGGCG
			R: GGTGGTGGTGGTGGTGGTGCTCGAGTGCG GCCGCAAGCTTTTTGTAGAGC
CurA- Halogenase	pET28b	N-poly(His)_6_	F: TCATGCCATATGCCAAAAACTATGA ACCGGGA
			R: TCATCGCTCGAGTTATTAGATGCTTG GTGTTTCC

1 F: Forward; R: Reverse.

### Cell-free Expression

Batch CF reactions were performed in 96 well V-shape microplates (PS-microplate 96 well V-shape, Greiner Bio-One, Frickenhausen, Germany) in a final reaction volume of 25 µl at a temperature of 30°C and with gentle shaking. The basic reaction mixture (RM) contained 2.5 mM ATP, 1.7 mM each of GTP, UTP and CTP, 34 mg/ml folinic acid, 170 µg/ml *E. coli* tRNA mixture (Roche, Penzberg, Germany), 4–15 ng/µl of plasmid template DNA, 10 µg/ml T7 RNA polymerase, 2 mM each of the 20 proteinogenic amino acids, 0.53 mM NAD^+^, 0.26 mM CoA, 280 mM K^+^-glutamate, 10 mM NH_4_^+^-glutamate, 10 mM Mg^2+^-glutamate, 1.5 mM spermidine, 1.5 mM putrescine, 4 mM Na^+^-oxalate, 1 mM DTT and 0.24% (v/v) of S30 extract in analytical scale reactions or 31% (v/v) in preparative scale reactions (Table 2) [5]. If Mg^2+^ ions were not analyzed as screening reagent, the final Mg^2+^ concentration of the reaction was adjusted to 26 mM with Mg^2+^-glutamate. The 10-fold premix prepared for screening reactions contained 15 mM putrescine, 15 mM spermidine, 2.5 M K^+^-glutamate, 100 mM NH_4_^+^-glutamate, 100 mM Mg^2+^-glutamate, 40 mM Na^+^-oxalate, 330 mM Na^+^-pyruvate, 340 µg/ml folinic acid, 10 mM DTT, 5.3 mM NAD^+^(Table 2). The premix could be stored at −20°C and refrozen multiple times without detectable loss of efficiency.

**Table 2:** CF reaction protocol for compound screening.

Compound	Stock	Final	Range
**Premix:**	**10-fold**	**1-fold**	
Putrescine	15 mM	1.5 mM	
Spermidine	15 mM	1.5 mM	
K^+^-glutamate	2500 mM	250 mM	
NH_4_^+^-glutamate	100 mM	10 mM	10–30 mM
Mg^2+^-glutamate	100 mM	10 mM^1^	
Na^+^-oxalate	40 mM	4 mM	
Na^+^-pyruvate	330 mM	33 mM	
Folinic acid	340 µg/ml	34 µg/ml	
DTT	10 mM	1 mM	
NAD^+^	5.3 mM	0.53 mM	
**Individual compounds:**			
20 amino acid mix	8 mM each	2 mM each	
PEP-K^+^	1 M	30 mM	
CoA-Na^+^	30 mM	0.26 mM	
*E. coli* tRNA	40 mg/ml	0.17 mg/ml	
T7-RNA-polymerase	1.4 mg/ml	10 µg/ml	5–10 µg/ml
NTP-Mix: ATP	90 mM	2.5 mM	
NTP-Mix: C/G/UTP (each)	60 mM	1.7 mM	
DTT	500 mM	optional	1–10 mM
Plasmid template	0.3 mg/ml	0.015 mg/ml	
*E. coli* S30 extract	100%	24% or 31%^2^	22–35%
Mg^2+^-glutamate	100 mM	16 mM^1^	20–30 mM^1^
H_2_O		fill up to 25 µl	

1 if not used as screening compound, the total final Mg^2+^ concentration was adjusted to 26 mM.

2 24% were used for analytical scale screening reactions, whereas 31% were used for preparative scale reactions.

### Compound Screening

Batch reactions were pipetted with a Tecan Freedom EVO 200 device equipped with an eight channel liquid handling arm (4×1,000 µl and 4×50 µl syringes) and two transport arms (Tecan, Männedorf/Zürich, Switzerland). The pipetting range was in between 300 nl and 800 µl. Stock solutions of chemicals (Sigma-Aldrich, Steinheim, Germany) were prepared in either H_2_O or 500 mM HEPES-KOH buffer, pH 8.2, and kept on cooling carriers at 4°C upon pipetting. All additives were adjusted prior addition to pH 8.2 by titration with either 500 mM HEPES-KOH, pH 8.2, or with 100 mM L-glutamic acid.

Linear concentration screening of selected single compounds as well as correlated concentration screening of two compounds was programmed by the custom designed EYES software based on the Gemini operating system. In a first step, the final concentration of each reaction compound was calculated and liquid classes for proper pipetting were defined. A mastermix of common compounds was then prepared and the screening compounds were pipetted first into the individual cavities of 96well microplates, followed by appropriate volumes of the mastermix. Processing time for calculation and pipetting was approximately 30–45 min per one complete 96well microplate screen. During pipetting, the microplate was chilled at 4°C and the reactions were started by addition of template DNA with subsequent incubation at 30°C on a shaker.

### Protein Quantification

Proteins containing red shifted sGFP fusions were quantified by fluorescence measurement with an excitation wavelength of 484 nm and emission wavelength of 510 nm [5]. Further method parameters were defined in the TECAN Magellan 5.03 software: Gain (Manual): 25; Number of reads: 10; Integration time: 40 µs; Lag time: 0 µs; Mirror selection: automatic; Multiple reads per well (Circle): 2×2; Incubation time: 20 s; Settle time: 20 s. Protein concentration was calculated from the measured sGFP fluorescence according to a calibration curve with purified sGFP. Potential effects of the analyzed chemicals on sGFP were determined by fluorescence measurements after incubating aliquots of 300 µg/ml purified sGFP with corresponding chemicals at 30°C for 4 hrs.

Alternatively, immunoblotting using anti-His antibodies or proteins labeled with ^35^S-methionine were used for quantification. ^35^S-methionine mixed with non-labeled amino acids in a ratio of 1∶40,000 were added into the reaction. After expression, samples were transferred into reaction tubes, centrifuged at 22,000×g for 10 min and the supernatant was precipitated with 10% trichloric acid. After washing, the pellet and the precipitated supernatant were measured for radioactivity. Control experiments without any DNA template were used as background value for the radioassay.

### Activity Assay of GNA1-sGFP

The 50 µl reactions were transferred into D-tubes (Novagen, Darmstadt, Germany), diluted with 50 µl buffer (50 mM Tris-HCl, pH 8.0) and dialyzed against 500 ml buffer with stirring at 4°C for 2 hrs. Samples were then centrifuged at 22,000×g for 10 min and supernatants were used for enzyme activity assay. The assay was performed in 50 µl buffer containing 500 mM D-glucosamine 6-phosphate (GlcN6P), 500 mM AcCoA, 50 mM Tris-HCl, pH 8.0, 5.0 mM MgCl_2_ and 10% glycerol in 96well flat bottom plates. Approximately 0.4 µg unpurified GNA1-sGFP (determined by fluorescence) were added to start the reaction. After incubation at 30°C for 5 min, the reaction was terminated by adding 50 µl of stop solution (50 mM Tris–HCl, pH 8.0, and 6.4 M guanidine hydrochloride) and then 50 µl of CR buffer (50 mM Tris–HCl, pH 8.0, 1 mM EDTA, and 200 µM 5,5′-dithiobis(2-nitrobenzoic acid) (DTNB). The amount of CoA produced by GNA1 was determined by 4-nitrothiophenolate formation and measured at 412 nm in a microplate reader (Fisher Scientific, Schwerte, Germany). A blank reaction using CF reactions without GNA1-sGFP template was used as control. The amount of CoA produced was calculated using the extinction coefficient of DTNB at 30°C (13,800 M^−1^ cm^−1^).

## Results and Discussion

### Basic CF Reaction Set Up for Robotic Screening Applications

The production of fluorescent sGFP was used as fast monitor for setting up the basic reaction protocol and for the subsequent evaluation of compound compatibility. In order to reduce pipetting time, a number of standard reaction compounds including salts, polyamines and some precursors were combined in a premix (Table 2). S30 extract, enzymes, unstable reagents and screening compounds were kept separately. The premix is stable at −80°C for at least one year and remains active after repeated freeze-thaw cycles [17]. Protein synthesis with the basic batch protocol is effective over 2 hrs and then reaches a plateau at production levels of approximately 0.5–0.8 mg sGFP per ml of batch reaction. Folding of sGFP is oxygen dependent and the plates were therefore further incubated for 2 hrs after the reaction prior to fluorescence determination.

**Figure 1:**
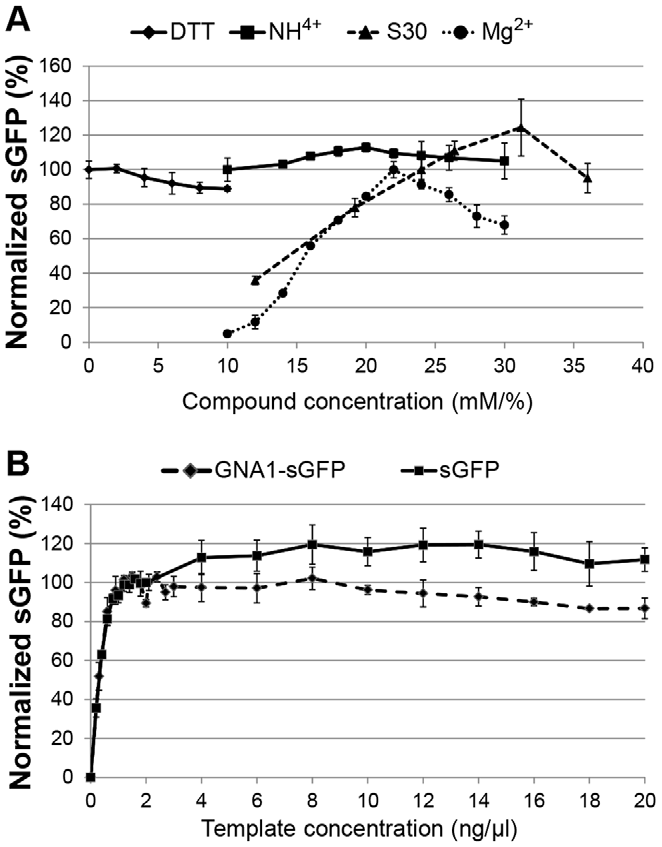
Linear concentration screens of basic CF batch reaction compounds. Expression efficiency was determined by sGFP fluorescence. A: Basic compounds S30 extract, DTT, NH_4_^+^, Mg^2+^; B: Plasmid templates.

Working lists for programming and pipetting were generated by the specific EYES software and optimal concentration ranges for several basic compounds were determined by linear or correlated concentration screening (Table 2). The S30 extract had a well-defined optimum at approximately 31% final concentration (Fig. 1A). Mg^2+^ ions are known to be critical for CF reactions and optimal concentration ranges were determined in between 20–28 mM depending on the S30 extract preparation. Reducing conditions could become important depending on the nature of the synthesized target proteins. DTT as reducing agent is tolerated in the reaction at least up to 10 mM final concentration while it could also be completely omitted without significant effects. NH_4_^+^ ions were tolerated at least up to 30 mM final concentration (Fig. 1A). Protein expression increased with plasmid DNA template concentrations up to 2–4 ng/µl reaction and then remained at a relatively stable plateau. The DNA template concentration optimum appeared to be independent from the coding regions of sGFP or GNA1-sGFP (Fig. 1B).

**Figure 2:**
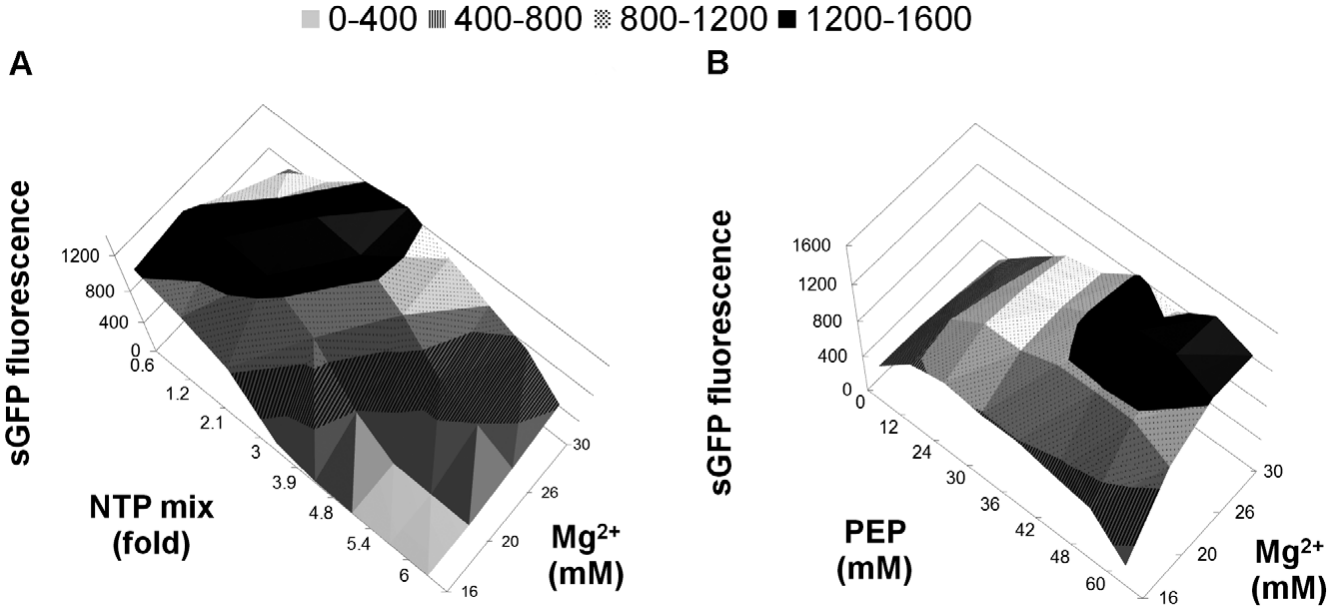
Correlated concentration screens with Mg^2+^ ions. Expression efficiency was determined by sGFP fluorescence. A: NTP mix/Mg^2+^; B: PEP/Mg^2+^.

Mg^2+^ ions could interact with other negatively charged compounds of the reaction such as NTPs or PEP and correlated optimal concentration ranges were analyzed (Fig. 2). With the combination of NTP mix and Mg^2+^, optimal efficiency was determined within the range of 1–2 fold NTP mix and 20–26 mM Mg^2+^ (Fig. 2A). With the combination of PEP and Mg^2+^, optimal concentrations were ranging from 36–50 mM and 24–30 mM, respectively (Fig. 2B). After establishing reaction conditions, the protein production in the CF batch reaction could be scaled up to at least 1 ml reaction volumes without loss of efficiency.

### PEG Derivatives as CF Additives

**Figure 3:**
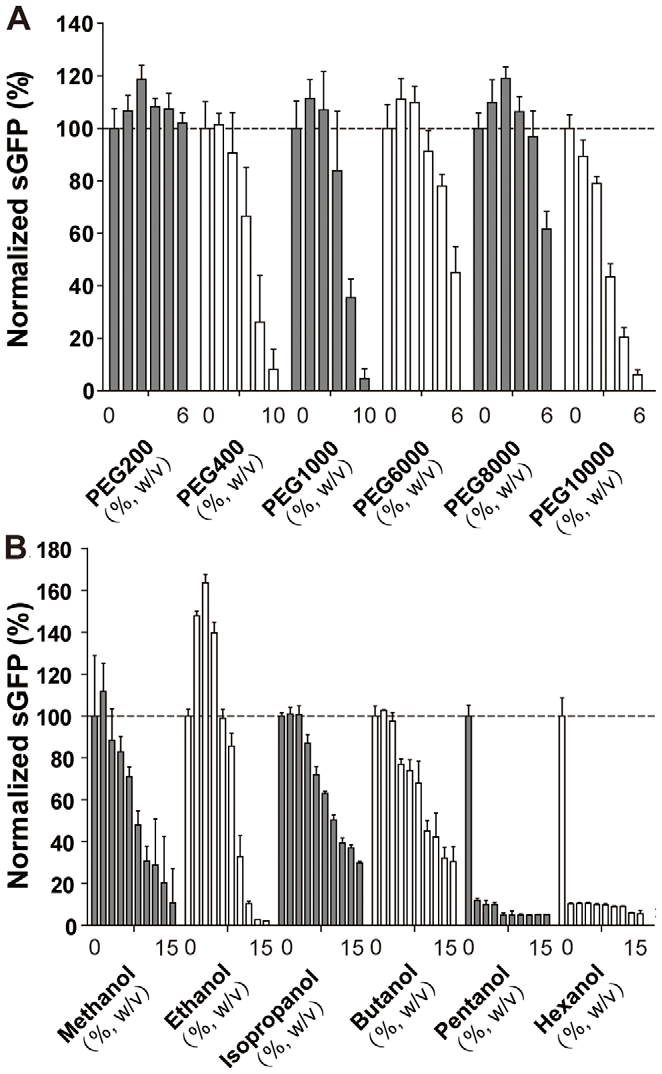
Effect of PEG and alcohols on fluorescent sGFP expression in the CF batch configuration. The first bar of each set indicates the control without added compound. Data are averages of at least three determinations. A: Screening of PEGs of different molecular weight. The sGFP protein control was 600–750 µg/ml. B: Effect of alcohols. The sGFP protein control was approximately 500 µg/ml.

PEG derivatives are known to act as molecular crowding agents by binding water thus making other reaction compounds more readily accessible. PEGs with increasing average molecular weights starting from 200 up to 8,000 kDa were added and with the exception of PEG 400 resulted into an increased sGFP fluorescence of 10–20% at final concentrations of 2–3% (Fig. 3A). The addition of PEG 10,000 resulted into an instant precipitation of reaction components presumably due to protein denaturation. PEG and other molecular crowding agents have been used to condense reactants and to mimic cellular environments in CF systems based on wheat germ extracts [18], [19]. A more detailed study revealed that PEG 8,000 resulted into increased CF transcription but rather reduced CF translation [18] and also different effects correlated with the PEG molecular weight on proteins are known [20]. However, systematic analysis of PEGs with different molecular weights in CF systems have not been made yet.

### Alcohols as CF Additives

Organic solvents are usually denaturizing by disrupting hydrophobic contacts in between the nonpolar side chains of amino acids. These effects are concentration dependent and some solvents such as alcohols or ketones can even act as protein stabilizers at lower concentrations while they convert to denaturants at high concentrations [21]. A further important parameter for stabilizing effects is the chain length of alcohols. We have analyzed alcohols of chain lengths from one to six carbon atoms for their compatibility with our CF system and for their effects on sGFP fluorescence (Fig. 3B). With the exception of ethanol, all other analyzed alcohols had concentration dependent negative effects on sGFP fluorescence most likely due to inhibition of factors essential for the basic protein expression machinery [22]. With pentanol and hexanol, already the lowest supplied concentration resulted in almost complete inhibition of sGFP expression and precipitate formation indicated substantial denaturation of proteins from the S30 extract. Addition of ethanol at 6–8% final concentration resulted into an 60% increase of sGFP fluorescence corresponding to an expression of approximately 800 µg/ml (Fig. 3B).

Our results are consistent with previous observations that denaturation effects of alcohols are correlated with their chain length and concentration. Low concentrations of ethanol in between 0.1–2.5% stabilized proteins and inhibited the mechanical denaturation of hemoglobin or the degradation of cytosolic proteins [23]. In the *E. coli* CF system, ethanol appears to be most promising in promoting protein expression as a result of either stabilizing the expression machinery and/or improving the folding of sGFP. Methanol, isopropanol and butanol had only minor positive effects but were tolerated to some extent up to 4–6% final concentration. Alcohols are frequently used in combination with detergents in order to stabilize hydrophobic membrane proteins in crystallization studies. The CF compatible alcohols might thus be considered as potential stabilizers of these protein types in future expression approaches.

### Natural Cellular Stabilizers as CF Additives

Living cells can produce a number of small molecules in order to stabilize intracellular proteins in extreme environmental conditions [10]. The major classes of these compounds are (i) polyols/sugars, (ii) amino acids and (iii) polyions. Polyols can protect proteins against a variety of denaturation and degradation mechanisms including aggregation, thermal denaturation, deamidation and oxidation [24], [25]. Further applications are preventing protein dehydration upon freeze-drying by serving as water substituent through hydrogen bonding. Sucrose and glycerol have become standard stabilizers for the long-term storage of protein samples. Protein protection by individual polyols can act in different ways and even mixtures might therefore be considered for optimal effects [26]. Amongst the most frequent polyols synthesized in various organisms are sucrose, glycerol, D-trehalose, D-mannose or D-sorbitol [27]. For lysozyme, D-mannitol was found to prevent aggregation, sucrose acted against deamidation and lactose reduced oxidation [28].

**Figure 4:**
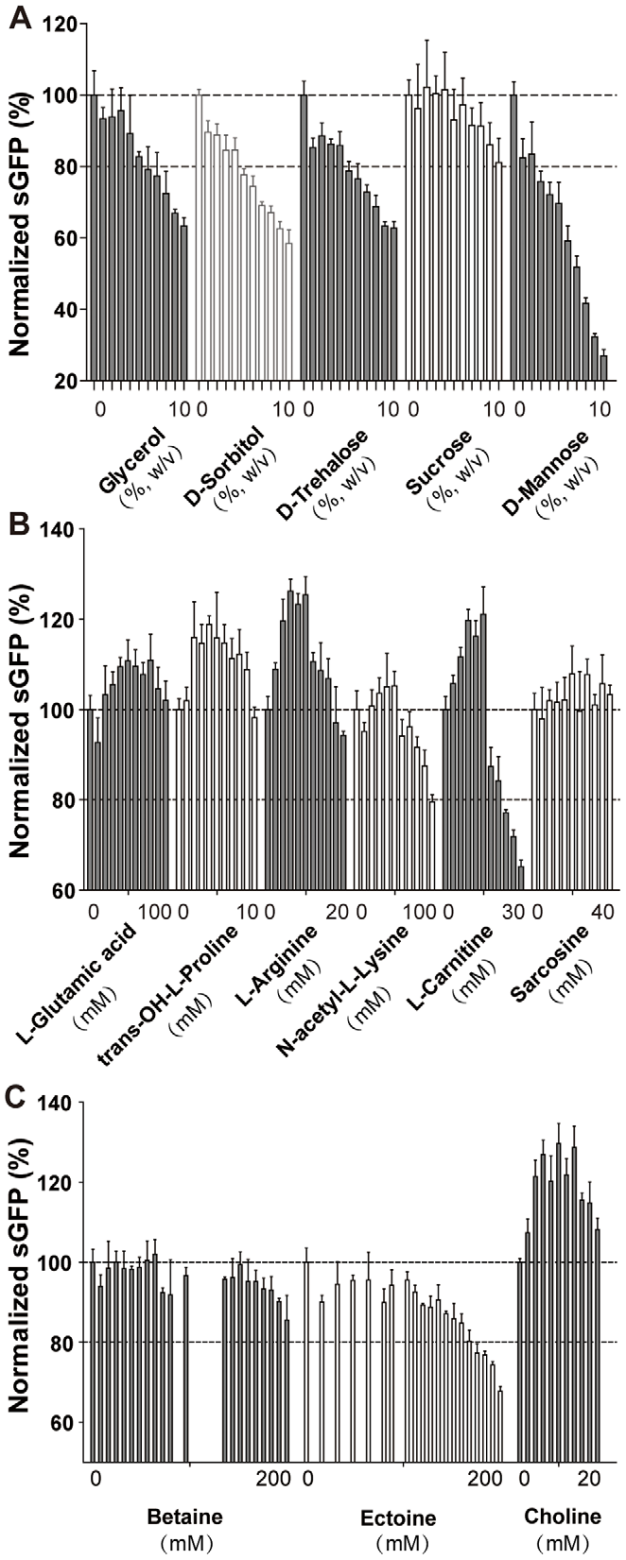
Effect of potential protein stabilizers on fluorescent sGFP expression in the CF batch configuration. The first bar of each set indicates the control without added compound and with sGFP production of approximately 500 µg/ml reaction. Data are averages of at least three determinations. A: Polyols; B: Amino acids; C: Polyions.

We have analyzed the compatibility of glycerol, sucrose, D-sorbitol, D-trehalose and D-mannose for our CF system by monitoring fluorescent sGFP expression (Table 3). D-sorbitol, D-trehalose and D-mannose were dose dependent inhibitors of fluorescent sGFP production starting already at 1% final concentration in the reaction (Fig. 4A). In contrast, sucrose and glycerol are tolerated up to 8% and 4% final concentration, respectively. Both compounds could thus be considered as potential CF additives in the determined tolerated concentration ranges.

**Table 3:** Compatibility of protein stabilizing compounds to the CF system.

Class	Compound	sGFP^1^	Working range^2^	Class	Compound	sGFP^1^	Working range^2^
**Polyions**	Betaine	±	>250 mM	**Amino acids**	L-OH- proline	++	>10 mM
	Choline	++	>20 mM		N-acetyl-L- lysine	+	<100 mM
	Ectoine	±	<150 mM		L-carnitine	++	<10 mM
**Polyols**	Sucrose	±	≤10%		L-arginine	++	>20 mM
	Glycerol	±	<8%		Sarcosine	+	>40 mM
	D-trehalose	±	<4%		L-glutamic- acid	++	>400 mM^3^
	D-mannose	±	<2%	**Alcohols**	Methanol	+	≤5%
	D-sorbitol	±	<4%		Ethanol	+	≤8%
**PEGs**	PEG 200	+	>6%		Isopropanol	±	≤5%
	PEG 400	±	<4%		Butanol	±	≤3%
	PEG 1,000	+	<6%		Pentanol	-	<1%
	PEG 6,000	+	<4.8%		Hexanol	-	<1%
	PEG 8,000	+	<4.8%	**Others**	PEI 2,000	-	<0.001%
	PEG 10,000	±	<1%		Urea	±	<100 mM

1 effect on fluorescent sGFP expression: ±, tolerated over a certain concentration range; -, decrease in fluorescent sGFP expression;+and ++, increase in fluorescent sGFP expression.

2 working range is defined with no more than 20% decrease in fluorescent sGFP expression. At the indicated concentration limits, the analyzed chemicals have either no effect or a slight quenching effect of maximal 10% on sGFP fluorescence.

3 used as basic buffer compound.

Amino acids can have a dual role in CF expression systems as they primarily serve as substrater for translation, but also could help to stabilize the expression machinery and/or the synthesized target protein. Proteinogenic amino acids such as L-arginine and L-glutamic acid in addition to some non-proteinogenic amino acids such as trans-OH-L-proline, N-acetyl-L-lysine and L-carnitine are known as protein stabilizers *in vitro* [29] and the concentration ranges compatible to the CF system were determined by fluorescent sGFP monitoring (Fig. 4B). Overall, all tested amino acids showed beneficial effects with some 10–20% increased sGFP fluorescence. The concentration optima were different and ranging from 50–80 mM for glutamic acid, 20–90 mM for trans-OH-L-proline, 20–50 mM for L-arginine, 30–50 mM for N-acetyl-L-lysine, 30–50 mM for L-carnitine and 50–70 mM for sarcosine. In particular N-acetyl-L-lysine and L-carnitine rapidly inhibit sGFP expression above their optimal concentrations while the concentration optima of the other amino acids have a more Gaussian appearance.

The polyions betaine, choline and ectoine are synthesized by organisms living in extremophile environments for the stabilization of cytoplasmic proteins. However, even *E. coli* is able to synthesize high amounts of betaine under some conditions [30]. Stabilizing effects have been shown with the inhibition of the *in vitro* insulin amyloid formation by ectoine or betaine [25]. For betaine and ectoine, a high tolerance of up to approximately 150 mM and 100 mM was determined in the CF system (Fig. 4C). However, neither compound had a positive effect on sGFP fluorescence. In contrast, an approximately 30% increased sGFP fluorescence was measured in presence of 4–14 mM choline. The general compatibility of choline was lower if compared with the two other polyions and below approximately 30 mM final concentration.

### Improving the Soluble CF Expression of Human GNA1 and of CurA Halogenase by Addition of Stabilizers

As a first proof of principle, we approached to improve the CF expression of two targets known to partly precipitate as aggregates. The human glucosamine 6-phosphate N-acetyltransferase (GNA1) is required for the *de novo* synthesis of N-acetyl-D-glucosamine-6-phosphate representing an essential precursor in UDP-GlcNAc biosynthesis [31]. The protein was synthesized with a C-terminal fusion to sGFP. The 40.5 kDa halogenase domain of the polyketide synthetase CurA from *Lynbya majuscula* was synthesized with a N-terminal poly(His)_6_-tag [16]. Efficient CF expression protocols for both enzymes have been established with yields exceeding 1 mg/ml. However, solubility is limited and approximately 30–50% of the expressed proteins precipitate during the reaction.

**Figure 5:**
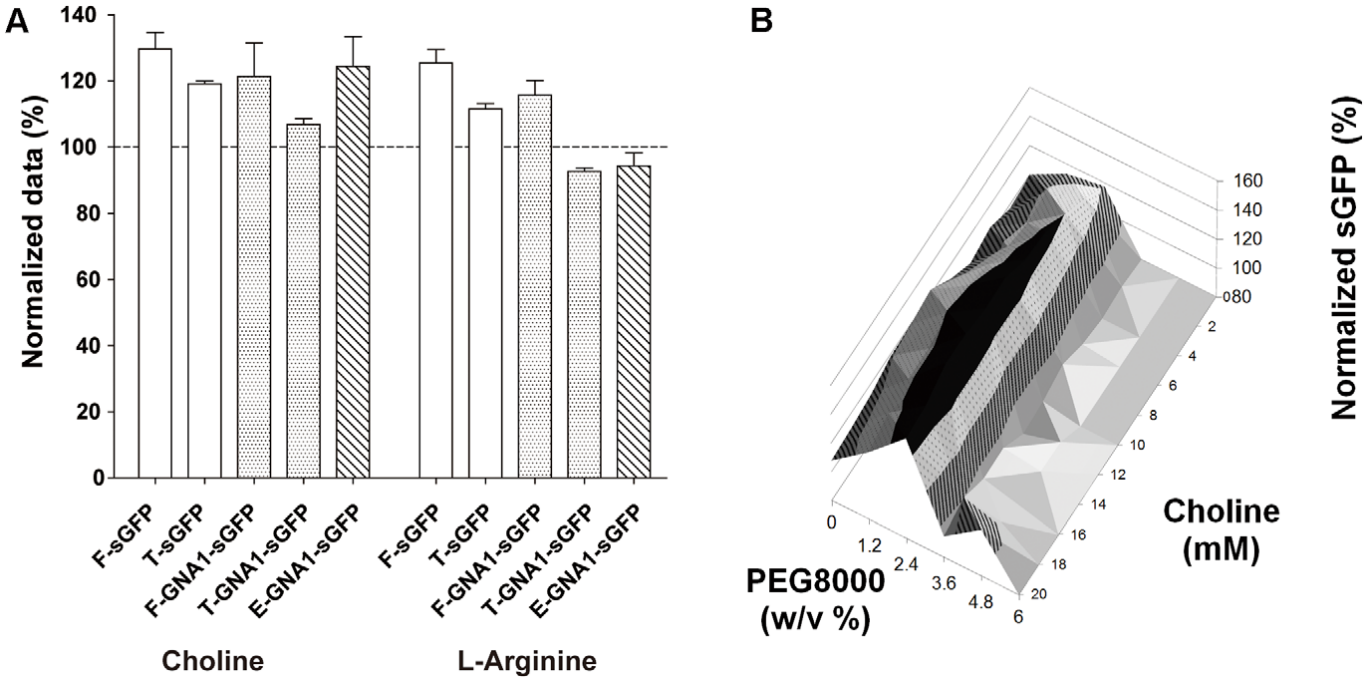
Effect of potential stabilizers on the quality of CF expressed sGFP and GNA1-sGFP. A: Choline or L-arginine were added at final concentrations of 10 mM each. Controls without any additives were taken as 100%. Soluble protein expression was measured by sGFP fluorescence, total protein production was quantified by ^35^S-Met incorporation and functional folding of GNA1 was analyzed by enzymatic activity. F, fluorescence; T, total protein production; E, enzymatic activity. B: Correlated screening of PEG 8,000 and choline for fluorescent expression of GNA1-sGFP. Controls without any additives were taken as 100%. Black, 160–180%; Dots, 120–160%; Lines, 80–120%; Gray, 0–80%.

Considering the screening results of the analyzed types of additives, only representative compounds shown to be tolerated by the CF system were analyzed for potential stabilizing effects on the two proteins. The addition of sucrose, D-sorbitol, ectoine or betaine in the tolerated concentration ranges had no effects on the soluble expression of GNA1-sGFP as monitored by sGFP fluorescence (data not shown). However, either 10 mM choline or 10 mM L-arginine increased the GNA1-sGFP fluorescence by approximately 20% (Fig. 5A). The addition of choline and L-arginine could either stabilize the general expression machinery resulting into higher yields, and/or they could stabilize the synthesized protein resulting in increased solubility. In order to investigate the reason for increased GNA1-sGFP fluorescence, the total protein production in the CF reaction was quantified by ^35^S-Met incorporation measurements. In addition, CF sGFP expression was included as a second reference reaction and the specific enzymatic activity of GNA1-sGFP was furthermore determined. The total sGFP expression as determined by ^35^S-Met incorporation was increased with either 10 mM L-arginine or 10 mM choline to 10% and 20%, respectively (Fig. 5A). However, in contrast with GNA1-sGFP only a slight increase with 10 mM choline was detectable while even a minor reduction of the total yield was measured with 10 mM L-arginine. Moreover, the increase in GNA1-sGFP fluorescence correlated with higher specific activity of the GNA1 enzyme upon addition of 10 mM choline into the CF reaction. Choline therefore appears to have multiple stabilizing effects in the CF expression reaction. The increased total protein production indicates a basic beneficial effect on the CF expression machinery that also at least partly contributes to the increased fluorescence of sGFP and GNA1-sGFP in the soluble protein fractions. However, an additional stabilizing effect of choline on the synthesized proteins is measured by the observed increased specific activity of GNA1. Accordingly, also the effect of L-arginine on sGFP fluorescence appeared to be cumulative based on higher expression as well as on better solubility. This is in accordance with previous observations of better folding of GFP in presence of L-arginine [32]. Interestingly, L-arginine increased solubility of GNA1-sGFP but not its total expression or specific activity. Therefore, even basic beneficial effects of stabilizers on the CF expression machinery appear to be template dependent and might be determined by improved formation of e.g. specific translation initiation complexes.

Choline and L-arginine as individual additives improved the CF production of soluble GNA1-sGFP for some 10–20%. We therefore analyzed whether beneficial compounds could have synergistic effects if added in a cocktail. Surprisingly, the combination of choline with L-arginine in correlated concentration screens was not cumulative and even some reduction in solubility was observed (data not shown). However, correlated screening of further stabilizer combinations identified a synergistic effect of choline with PEG 8,000, resulting in 50–60% increased fluorescent GNA1-sGFP production when a concentration range of 8–16 mM choline and 2–3% PEG 8,000 was used (Fig. 5B). This result demonstrates that effects of stabilizer combinations are hard to predict and underlines the need for a systematic screening approach.

**Figure 6:**
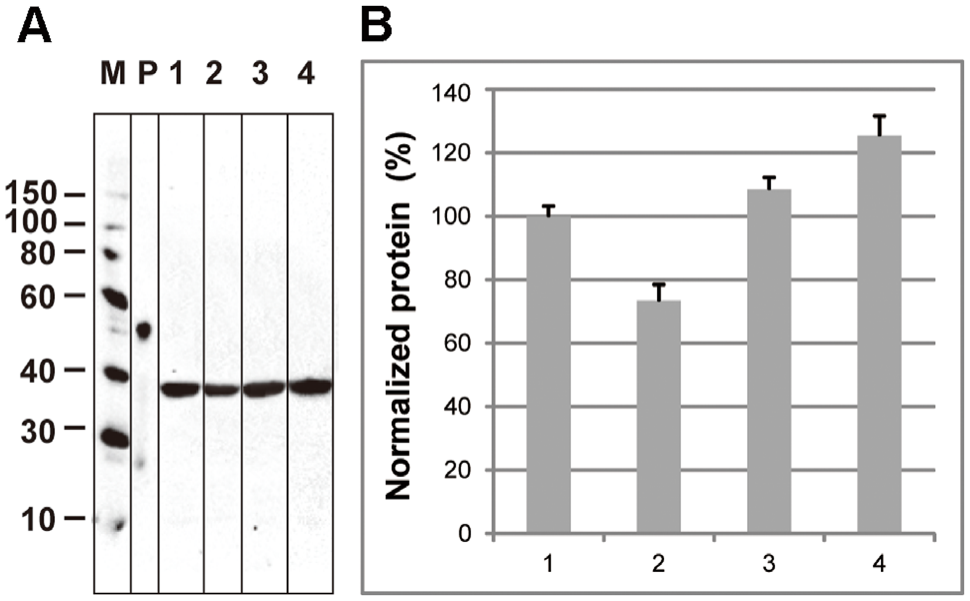
Effect of protein stabilizers on the soluble expression of CurA halogenase. The CurA halogenase domain was expressed in the batch configuration with different additives. Protein production was quantified by immunoblotting. The results were normalized with the control as 100% corresponding to a protein concentration of 80 ng/µl. A: Immunoblot with anti-penta-His antibody. M, marker proteins in kDa; P, positive control for quantification (Positope™, invitrogene). B: Quantification of band intensity. 1, control; 2, 6% D-trehalose; 3, 10 mM L-arginine; 4, 10 mM choline.

As a further target, the soluble CF expression of the halogenase domain of CurA was analyzed (Fig. 6). The reactions were supplemented with either 10 mM choline, 10 mM L-arginine or 6% D-trehalose and the protein in the supernatant was quantified after the reaction by immunoblotting. In accordance to the results obtained with sGFP, the addition of L-arginine and choline again resulted into 8% and 25% increased soluble expression, while the presence of D-trehalose was inhibitory.

### Conclusions

Small molecules belonging to different groups of natural chemical chaperones can be added into CF expression reactions and acting as general or specific stabilizers. This work has defined the working ranges in CF expression systems for a representative variety of the most commonly employed chemical chaperones. The tolerated concentrations of the supplied chemicals by the CF system are different from those reported from living organisms and a number of compounds tolerated *in vivo* became rapidly inhibitory to the CF expression machinery. As most promising stabilizing agents for the analyzed proteins we could define ethanol, PEG derivatives, amino acids and choline. However, additional polyols and polyions are also tolerated at relatively high concentrations and might therefore be useful in expression approaches with other target proteins. We could show that stabilizing effects can depend on the nature of the target protein as well as on the combination of several additives. Modes of action of the analyzed stabilizers include increased expression, better solubility as well as improved stability and could be exclusive or cumulative. We therefore propose and have established an empirical screening approach in order to define the optimal concentration balance of stabilizers in individual CF protein expression approaches. The presented CF screening platform will become accessible to the scientific community in the European INSTRUCT network (www.structuralbiology.eu).
